# Evaluation of the Quality of Antibiotic Prescribing in Primary Care: A Multicenter Longitudinal Study From Shenzhen, China

**DOI:** 10.3389/fphar.2020.617260

**Published:** 2021-02-19

**Authors:** Yanhong Gong, Hui Li, Heping Yang, Kun Tan, Wei Liu, Xiaotong Li, Jianxiong Wu, Guopeng Zhang, Xiaoxv Yin

**Affiliations:** ^1^ Department of Social Medicine and Health Management, School of Public Health, Huazhong University of Science and Technology, Wuhan, China; ^2^ School of Nursing, Wuchang University of Technology, Wuhan, China; ^3^ Department of Infection Control, Tongji Hospital, Tongji Medical College, Huazhong University of Science and Technology, Wuhan, China; ^4^ Department of Public Health, Tongji Hospital, Tongji Medical College, Huazhong University of Science and Technology, Wuhan, China; ^5^ Department of Nuclear Medicine, Tongji Hospital, Tongji Medical College, Huazhong University of Science and Technology, Wuhan, China

**Keywords:** antibiotic prescribing, quality evaluation, primary care, antibiotic stewardship, longitudinal study, China

## Abstract

**Background:** Currently, there is no comprehensive evaluation of the quality of antibiotic prescribing in China’s primary care facilities based on longitudinal data.

**Methods:** We randomly selected 11 community health centers in Shenzhen, China, and collected all outpatient prescriptions of these centers from 2010 to 2015. To evaluate the quality of antibiotic prescribing, we used six quality indicators for analysis, including number of antibiotics per 100 consultations, ratio between broad-spectrum and narrow-spectrum antibiotics (B/N ratio), percentage of first-line antibiotics recommended by guidelines, percentage of oral antibiotics with a duration exceeding the guideline recommendation, and new pediatric-specific indicators such as percentage of antibiotics with amoxicillin (A index) and ratio between amoxicillin and broad-spectrum antibiotics (A/B ratio).

**Results:** During the study period, 571,362 outpatient consultations resulted in antibiotic prescriptions, which contained 706,411 antibiotics. The overall number of antibiotics per 100 consultations decreased significantly from 93.50 in 2010 to 19.98 in 2015 (*p* = 0.004), but the B/N ratio showed an upward trend over time (*p* = 0.009). In different populations and different common infections, the number of antibiotics used decreased to varying degrees, while the B/N ratio increased to varying degrees, with the most obvious change in children <5 years. The percentage of first-line antibiotics for common infections was not high, ranging from 3.45 to 44.25% during 2014–2015. The percentage of oral antibiotics with an exceeded duration ranged from 0.70 to 19.39%. Moreover, the A index and A/B ratio in children remained low for a long time, which was 0.76% and 0.01 in 2015.

**Conclusion:** A review of antibiotic prescribing in Shenzhen, China, showed a substantial reduction in antibiotic use in primary care. However, problems such as widespread use of broad-spectrum antibiotics, insufficient use of first-line antibiotics and low use of amoxicillin were prevalent. Improving and optimizing the quality of antibiotic prescribing, particularly in children prescriptions, will be the focus of future antibiotic stewardship in China.

## Introduction

Excessive and inappropriate use of antibiotics not only caused a huge economic burden to global health systems, but also rapidly increased the risk of antimicrobial resistance (AMR). Many countries, including China, are trying to tackle global AMR by reducing unnecessary or inappropriate prescribing (Sabuncu et al., 2009; Smieszek et al., 2018; Xiao, 2018). Crucially, identifying these antibiotic prescribing is a necessary precondition for the implementation of national policy interventions and an important feedback for the evaluation of intervention effects.

Reliable quality indicators are needed to identify the quality of antibiotic prescribing. A common methodology for collecting and reporting national antimicrobial consumption data developed by the WHO program of surveillance provided information on the level of use and types of antimicrobials to prescribers (World Health Organization, 2016). The quality indicators developed by the European Surveillance of Antimicrobial Consumption to assess outpatient antibiotic use provided a basis for policy-makers to formulate interventions to address identified problems (World Health Organization Regional Office for Europe, 2017). Furthermore, the use of European quality indicators in Belgium, the Netherlands, Sweden, and the United Kingdom (UK) was feasible and won opportunities for quality improvement of antibiotic prescribing in primary care (de Bie et al., 2016; Tyrstrup et al., 2017; Robertson et al., 2018). These studies on quality evaluation in these countries provided an effective reference for China.

Abuse of antibiotic prescribing in China remains serious, particularly in primary care facilities (Yin et al., 2013; Wang et al., 2014; Liu et al., 2019). The medical visits to primary care facilities accounted for more than half of the total medical visits nationwide in 2017 (China National Health Commission, 2018). However, around 70% of outpatients attending these facilities were reported inappropriate antibiotic prescribing practices, such as overuse of antibiotics, widespread use of broad-spectrum antibiotics, and unnecessary prolonged treatment (Wang et al., 2014; Wei et al., 2017). In the past 2 decades, the Chinese government has made great efforts to promote the appropriate use of antibiotics (Xiao, 2018; National Health Commission of the People’s Republic of China, 2005; Xiao et al., 2013). In 2004, the Chinese Ministry of Health issued the *Guidelines for Clinical Application of Antimicrobial Agents in China*. In 2011, a long-term national antimicrobial stewardship campaign was launched nationwide. In 2012, China also implemented the strictest regulation yet for antibiotic stewardship, the *Administrative Measures for Clinical Use of Antimicrobial Agents*. Previous studies showed that overuse of antibiotics in China’s medical institutions at all levels have been significantly controlled (Lin et al., 2016; Li et al., 2019; Li et al., 2020). However, these studies mainly focused on the change of the frequency or amount of antibiotics. For the conformity of drug type and the appropriateness of treatment time, such evaluation report on prescription quality is currently blank. Moreover, it is noteworthy that the greatest source of antibiotic prescribing for outpatients in primary care is common infections, such as acute respiratory tract infections, acute otitis media, and gastroenteritis, etc. (Pouwels et al., 2019). And one-fourth of the outpatients receiving antibiotics for common infections are children (Dong et al., 2008). A comprehensive evaluation is also urgently needed to identify the quality of antibiotic prescribing for specific diseases and specific populations in these facilities.

Therefore, based on the longitudinal prescription data from community health centers in Shenzhen, China and referring to Chinese guidelines, this study evaluated the quality of antibiotic prescribing in primary care facilities from various aspects to provide a clear reference for antibiotic policy interventions in China.

## Materials and Methods

### Ethical Approval

This study was approved by the Research Ethics Committee of Tongji Medical College, Huazhong University of Science and Technology. Waiver for informed consent was granted by the ethics committees as no patients were involved in developing the research design or measurement of the outcome indicators.

### Study Design and Data Source

We retrospectively analyzed the consultation prescriptions of outpatients in primary care facilities in Shenzhen, China, from January 2010 to December 2015. Shenzhen, a metropolis with 13 million permanent populations, is located in southern China and has relatively developed primary care facilities, named as community health centers (CHCs).

The data were obtained from electronic information systems of CHCs in Xixiang Subdistrict, Shenzhen. Over the study period, there were 33 CHCs in Xixiang Subdistrict. We selected 11 from the 33 CHCs through a simple random sampling method to cover approximately 30% of patients (Li et al., 2020). Then we collected all outpatient prescription data for six consecutive years from their electronic information systems. In this database, each prescription included information on patient sex and age, disease diagnoses, medication costs, and details of prescribed medications (generic name; unit cost; numbers, dosage, and route of administration; treatment days). The *International Statistical Classification of Diseases, Tenth Revision (ICD-10)* codes was used to classify and code disease diagnoses (World Health Organization, 2014). The antibiotics were identified using the *Anatomic Treatment and Chemical (ATC) classification*, code J01 (World Health Organization, 2018).

### Indicators and Definition

We first adopted four quality indicators including number of antibiotics per 100 consultations, ratio between broad-spectrum and narrow-spectrum antibiotics (B/N ratio), percentage of first-line antibiotics recommended by guidelines and percentage of oral antibiotics with a duration exceeding the guideline recommendation to evaluate the quality of antibiotic prescribing in the overall populations (World Health Organization Regional Office for Europe, 2017; Pouwels et al., 2019). Then, taking into account differences in the prevalence of infection and drug use between children and adults, we used two child-specific indicators including percentage of antibiotics with amoxicillin (A index) and ratio between amoxicillin and broad-spectrum antibiotics (A/B ratio) to further evaluate the use of amoxicillin in children (de Bie et al., 2016). In this study, the broad-spectrum antibiotics included broad-spectrum penicillins, second- and third-generation cephalosporins and macrolides [J01CR, J01DC, J01DD and J01F (without erythromycin)]; the narrow-spectrum antibiotics included narrow-spectrum penicillins, first-generation cephalosporins and erythromycin (J01CE, J01DB and J01FA01) (de Bie et al., 2016). Our reference guideline was the *Guidelines for Clinical Application of Antimicrobial Agents in China* (2004 edition).

In China, a patient is typically prescribed one prescription per outpatient consultation. A single prescription contains one or more drugs. The number of antibiotics (J01) per 100 consultations was used to reflect the amount of antibiotics consumed by outpatients. The B/N ratio was expressed as the ratio between the number of broad-spectrum antibiotics used and that of narrow-spectrum antibiotics. The smaller the B/N ratio, the most appropriate the prescribing. The percentage of first-line antibiotics recommended by guidelines was expressed as the percentage of the number of first-line antibiotics used over the total number of antibiotics. The percentage of oral antibiotics with a duration exceeding the guideline recommendation was expressed as the percentage of the number of oral antibiotics with an exceeded duration over the total number of oral antibiotics. So the higher the percentage of first-line antibiotics or the lower the percentage of oral antibiotics with an exceeded duration, the most appropriate the prescribing. Furthermore, the A index was defined as the percentage of the number of amoxicillin (J01CA04) used over the total number of antibiotics. The A/B ratio was defined as the ratio between the number of amoxicillin used and those of broad-spectrum antibiotics. Thus, the greater the A index or A/B ratio, the most appropriate the amoxicillin prescribing for children.

### Data Analysis

In calculating percentage of first-line antibiotics and percentage of oral antibiotics with an exceeded duration, we included the 12 common infections in Chinese guidelines, including acute sinusitis, acute sore throat, acute cough and bronchitis, community-acquired pneumonia, acute otitis media, gastroenteritis, pericoronitis, acute periapical abscess, pelvic inflammatory disease, bacterial vaginosis, acute prostatitis, cellulitis (Supplementary Table S1). These infections came from the body systems of respiratory, digestive, genitourinary, or skin, and were common primary diagnoses for which antibiotics were generally prescribed in outpatients. The Chinese guidelines had been used for a long time since issued in 2004, and recommended the types of first-line antibiotics for common infections and the duration of antibiotic treatment, so as to regulate the rational use of antibiotics by clinicians. We further evaluated the quality of antibiotic prescribing for each of 12 common infections. Due to the lack of first-line antibiotics recommended and duration of treatment for four common infections (acute cough and bronchitis, community-acquired pneumonia, gastroenteritis, cellulitis) in the Chinese guidelines, we referred to relevant contents from the Public Health England guidance during 2013–2015 which was widely used (Pouwels, et al., 2019). For the analysis of treatment days, we included only oral antibiotics.

To explore the quality of antibiotic prescribing in different populations, particularly in children, we performed a stratified analysis by age group (<5, 5–17, and ≥18 years). To analyze trends in the quality of antibiotic prescribing, we combined all available annual data into three 2-year time periods (2010–2011, 2012–2013, and 2014–2015) and reported the absolute changes in quality indicators during 2010–2011 vs. 2014–2015. Moreover, we also calculated the total number of excess antibiotic days for 12 common infections, defined as the cumulative number of days beyond the recommended duration in the guidelines. We used linear regression analysis to test linear trends of indicators over time. The confidence interval (CI) was calculated using a logit transformation based on the estimated standard error. All statistical tests were two sided, and *p* values <0.05 were considered significant. All statistical analyses were performed using SAS version 9.4 (SAS Institute, Cary, NC, United States).

## Results

### Characteristics of Antibiotic Prescribing

Between 2010 and 2015, 1,482,223 outpatient consultations were conducted from 11 participating CHCs. Of these, 571,362 (38.55%) consultations led to antibiotic prescriptions, which contained 706,411 antibiotics. Among all antibiotic prescriptions, 79,496 (13.91%) antibiotic prescriptions were issued in young children <18 years. And the top three systematic diagnoses for antibiotic prescriptions were the diseases of respiratory, digestive, and genitourinary systems, accounting for 69.54, 9.84, and 8.49%, respectively. Among all antibiotics, the frequencies of oral antibiotics used were 337,208 (47.74%). A total of 90% of antibiotics were in turn covered by cephalosporins, macrolides, aminoglycosides, quinolones, imidazoles, and penicillins, involving 23 antibiotic classes. See Table 1 for details.

**TABLE 1:** Characteristics of antibiotic prescribing of outpatient consultations, 2010–2015 [n (%)].

Characteristics	2010–2011	2012–2013	2014–2015	Total
Total antibiotic prescriptions	305,511	157,067	108,784	571,362
Patient sex				
Male	155,807 (51.00)	75,614 (48.14)	52,947 (48.68)	284,368 (49.77)
Female	149,716 (49.00)	81,450 (51.86)	55,828 (51.32)	286,994 (50.23)
Patient age (years)				
<5	14,836 (4.86)	6,705 (4.27)	4,749 (4.37)	26,290 (4.60)
5–17	23,313 (7.63)	15,922 (10.14)	13,971 (12.84)	53,206 (9.31)
≥18	267,362 (87.51)	134,440 (85.59)	90,064 (82.79)	491,866 (86.09)
ICD10-diagnosis category				
Diseases of the respiratory system	231,474 (75.77)	103,428 (65.85)	62,413 (57.37)	397,315 (69.54)
Diseases of the digestive system	24,699 (8.08)	16,631 (10.59)	14,886 (13.68)	56,216 (9.84)
Diseases of the genitourinary system	16,950 (5.55)	16,990 (10.82)	14,572 (13.40)	48,512 (8.49)
Diseases of the skin and subcutaneous tissue	6,342 (2.08)	3,790 (2.41)	3,065 (2.82)	13,197 (2.31)
Symptoms, signs and abnormal clinical and laboratory findings	4,316 (1.41)	3,433 (2.19)	2,712 (2.49)	10,461 (1.83)
All other diagnoses	21,730 (7.11)	12,795 (8.15)	11,136 (10.24)	45,661 (7.99)
Total antibiotics	393,996	187,279	125,136	706,411
Administrational route				
Parenteral	189,524 (48.10)	88,317 (47.16)	62,211 (49.71)	340,052 (48.14)
Oral	178,235 (45.24)	96,053 (51.29)	62,920 (50.28)	337,208 (47.74)
Other	26,237 (6.66)	2,909 (1.55)	5 (0.01)	29,151 (4.13)
Antibiotic classification (J01 for systemic use)				
Other β-lactam antibacterials (J01D)	133,335 (33.84)	70,049 (37.40)	45,358 (36.25)	248,742 (35.21)
Macrolides, lincosamide and streptogramins (J01F)	122,074 (30.98)	45,672 (24.39)	20,165 (16.11)	187,911 (26.60)
Aminoglycoside antibacterials (J01G)	41,398 (10.51)	15,253 (8.14)	21,764 (17.39)	78,415 (11.10)
Quinolone antibacterials (J01M)	47,259 (11.99)	16,392 (8.75)	14,355 (11.47)	78,006 (11.04)
Imidazole derivatives of other antibacterials (J01XD)	36,013 (9.14)	24,445 (13.05)	13,154 (10.51)	73,612 (10.42)
β-lactam antibacterials, penicillins (J01C)	10,776 (2.74)	15,070 (8.05)	10,029 (8.01)	35,875 (5.08)
other antibiotics in J01	3,141 (0.80)	398 (0.22)	311 (0.25)	3,850 (0.55)

### Evaluation of the Quality of Overall Antibiotic Prescribing

**FIGURE 1:**
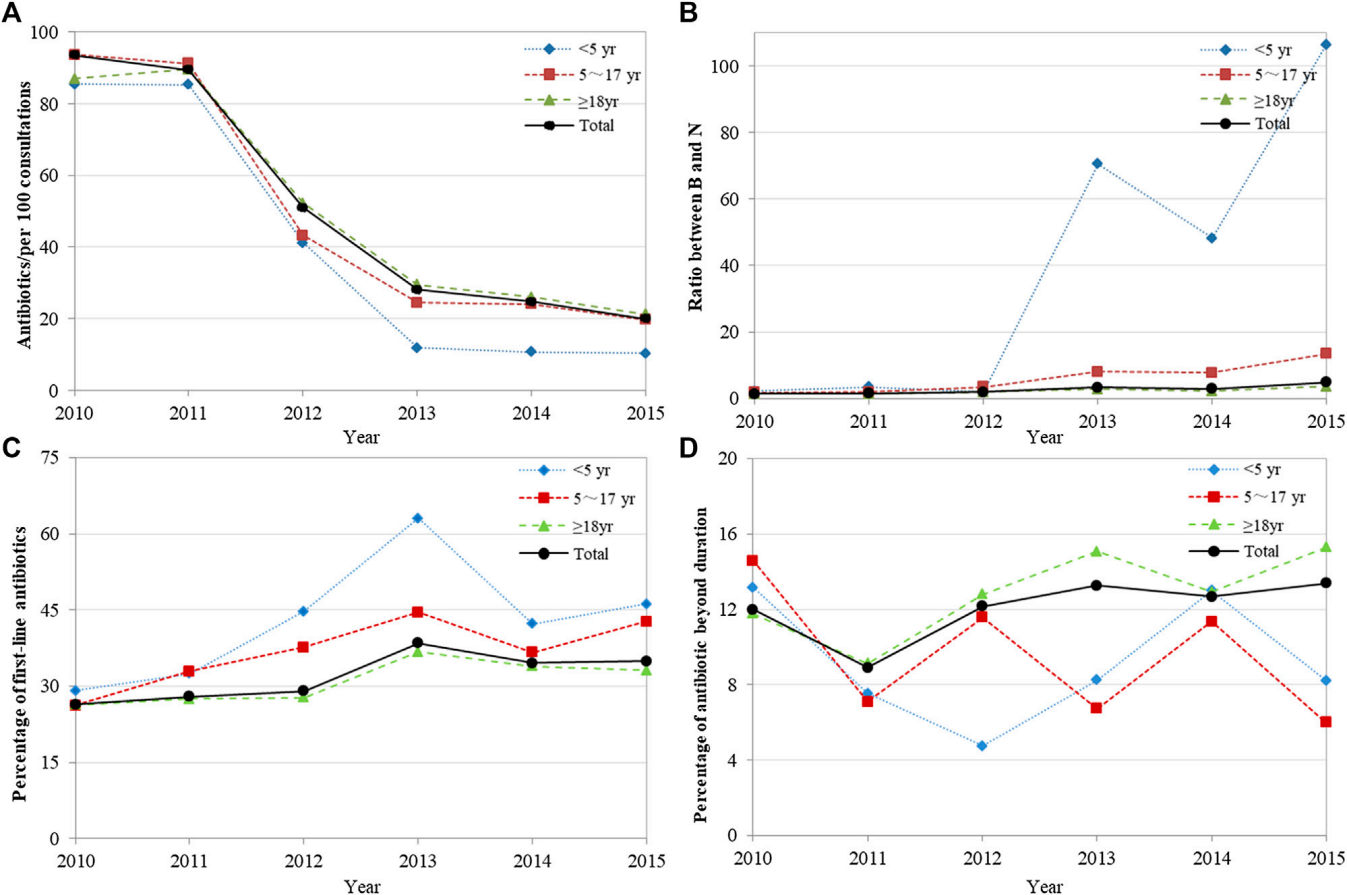
Changes in the quality of overall antibiotic prescribing, 2010–2015. **(A)** Antibiotics/per 100 consultations; **(B)** Ratio between B and N; **(C)** Percentage of first-line antibiotics recommended by guidelines; **(D)** Percentage of oral antibiotics with a duration exceeding the guideline recommendation. Ratio between B and N = Ratio between broad-spectrum and narrow-spectrum antibiotics.

The analysis of overall antibiotic prescribing showed that the number of antibiotics per 100 consultations decreased significantly from 93.50 in 2010 to 19.98 in 2015 (*p* = 0.004). The number of antibiotics used in different age groups decreased to different degrees, and the decrease was most evident in children aged <5 years, from 85.47 in 2010 to 10.37 in 2015 (*p* = 0.009) (Figure 1A). The overall B/N ratio showed an increasing trend over time (*p* = 0.009), and the increase was also most evident in children aged <5 years (*p* = 0.020) (Figure 1B). Referring to the Chinese guidelines, the overall percentage of first-line antibiotics for 12 common infections increased from 26.42% in 2010 to 34.91% in 2015, but the trend change was not statistically significant (*p* = 0.054) (Figure 1C). The percentage of first-line antibiotic used in different age groups first increased and then decreased, and the trend change was not obvious. In addition, the overall percentage of oral antibiotics with an exceeded duration for 12 common infections increased from 11.99% in 2010 to 13.38% in 2015. However, the trend change was not statistically significant (*p* = 0.181) (Figure 1D). Detailed data were shown in Supplementary Table S2.

### Evaluation of the Quality of Antibiotic Prescribing for Common Infections

During the study period, the total number of antibiotics used for the 12 included infections was 337,859. The results of different infections showed that except for acute periapical abscess (*p* = 0.198), the number of antibiotics used for other infections decreased to varying degrees. Acute otitis media decreased the most, with the number of absolute antibiotic changes per 100 consultations being −93.73 between 2010–2011 and 2014–2015 (*p* < 0.001) (Table 2). The B/N ratio of seven infections involving the systems of respiratory, digestive, and genitourinary showed different degrees of increase, and the B/N ratio of bacterial vaginosis in women was the highest by 2014–2015 at 18.34 (95% CI: 13.12 to 23.55) (Table 2). Moreover, the total number of first-line antibiotics recommended for 12 included infections was 103,787 (30.72% of total). The results of different infections showed that the percentages of first-line antibiotics recommended by guidelines ranged from 3.45 to 44.25% by 2014–2015. Although the percentages of first-line antibiotics used in acute sore throat, gastroenteritis, and pelvic inflammatory disease in women increased significantly by 6.22, 16.42, and 8.34%, respectively, the percentages of first-line antibiotics in these infections were still less than 50% (Table 2). The total number of oral antibiotics used for these 12 infections was 154,917, and 17,939 (11.58%) were oral antibiotics with an exceeded duration. The percentages of oral antibiotics with an exceeded duration for different infections ranged from 0.70 to 19.39% by 2014–2015, and this translated into 51,051 excess antibiotic days for these 12 infections. Despite a significant decline of 8.16% in this percentage of gastroenteritis (*p* = 0.026), 19.39% (95% CI: 18.32–20.49%) was still found in this percentage of acute cough and bronchitis (Table 2).

**TABLE 2:** Change in the quality of antibiotic prescribing for common infections, 2010–2015.

Indicators	2010–2011	2012–2013	2014–2015	*P* for overall linear trend^a^	Change value^b^
Antibiotics/per 100 consultations					
Acute sinusitis	130.86 (115.72–146.00)	58.40 (45.68–71.12)	43.73 (38.71–48.74)	<0.001	−87.13
Acute sore throat	107.15 (104.45–109.84)	40.09 (32.93–47.25)	24.16 (22.07–26.25)	<0.001	−82.99
Acute cough and bronchitis	116.89 (111.56–122.22)	46.84 (39.82–53.86)	28.11 (25.30–30.91)	0.005	−88.78
Community acquired pneumonia	129.36 (123.11–135.60)	83.20 (71.71–94.70)	48.68 (40.34–57.03)	0.002	−80.68
Acute otitis media	151.49 (145.39–157.60)	94.72 (82.42–107.03)	57.76 (52.69–62.82)	<0.001	−93.73
Gastroenteritis	87.72 (85.65–89.79)	35.72 (29.43–42.01)	20.56 (18.59–22.53)	0.007	−67.16
Pericoronitis	168.03 (164.22–171.84)	99.68 (88.96–110.40)	68.79 (65.97–71.62)	0.003	−99.24
Acute periapical abscess	168.88 (155.37–182.40)	112.33 (96.11–128.56)	134.83 (116.72–152.95)	0.198	−
Pelvic inflammatory disease	126.91 (118.49–135.33)	72.97 (64.89–81.04)	46.10 (41.51–50.70)	0.006	−80.81
Bacterial vaginosis	66.96 (61.63–72.29)	48.13 (42.90–53.36)	28.83 (25.46–32.19)	0.012	−38.13
Acute prostatitis	129.24 (111.60–46.88)	65.67 (53.17–78.17)	36.39 (28.43–44.34)	0.005	−92.85
Cellulitis	156.31 (121.97–190.65)	152.08 (118.56–185.59)	84.03 (72.99–95.07)	<0.001	−72.28
Ratio between B and N					
Acute sinusitis	2.66 (1.31–4.02)	2.26 (1.54–2.98)	3.84 (2.67–5.00)	0.117	−
Acute sore throat	1.54 (1.42–1.66)	2.12 (1.46–2.77)	4.19 (3.54–4.85)	<0.001	+2.65
Acute cough and bronchitis	2.20 (1.97–2.42)	4.10 (3.52–4.68)	6.50 (5.22–7.78)	0.016	+4.30
Community acquired pneumonia	7.29 (3.71–10.87)	4.96 (2.26–7.65)	11.47 (7.36–15.57)	0.089	−
Acute otitis media	1.06 (0.87–1.25)	1.86 (1.48–2.23)	3.85 (2.54–5.15)	<0.001	+2.79
Gastroenteritis	2.04 (1.63–2.44)	4.55 (3.00–6.10)	7.64 (5.96–9.33)	<0.001	+5.60
Pericoronitis	0.84 (0.70–0.98)	1.45 (1.16–1.75)	2.47 (1.71–3.23)	0.046	+1.63
Acute periapical abscess	2.33 (1.39–3.26)	2.77 (1.59–3.96)	3.84 (2.44–5.23)	0.009	+1.51
Pelvic inflammatory disease	13.7 (6.00–21.40)	18.35 (4.85–31.84)	15.79 (9.63–21.95)	0.602	−
Bacterial vaginosis	4.66 (3.03–6.29)	17.68 (7.99–27.37)	18.34 (13.12–23.55)	0.043	+13.68
Acute prostatitis	3.79 (1.34–6.24)	7.94 (5.59–10.29)	12.56 (−5.25–30.25)	0.112	−
Cellulitis	2.77 (−2.79–8.34)	3.88 (2.18–5.57)	7.41 (3.86–10.95)	0.226	−
Percentage of first-line antibiotics^c^					
Acute sinusitis	28.54 (7.97–49.11)	39.25 (13.12–65.37)	38.73 (29.61–47.85)	0.173	−
Acute sore throat	35.74 (33.94–37.53)	43.58 (40.79–46.36)	41.96 (38.88–45.03)	0.002	+6.22
Acute cough and bronchitis	4.10 (3.34–4.85)	4.26 (1.80–6.71)	4.06 (2.35–5.77)	0.973	−
Community acquired pneumonia	5.61 (0.56–10.66)	19.33 (6.94–31.71)	3.45 (1.32–8.22)	0.932	−
Acute otitis media	32.11 (26.53–37.70)	33.05 (28.01–38.08)	41.96 (29.51–54.42)	0.093	−
Gastroenteritis	19.06 (17.40–20.73)	28.29 (22.59–33.98)	35.48 (29.11–41.85)	<0.0001	+16.42
Pericoronitis	34.84 (32.04–37.64)	43.83 (41.06–46.61)	41.71 (39.01–44.42)	0.348	−
Acute periapical abscess	35.75 (28.54–42.96)	51.80 (36.95–66.66)	29.11 (23.67–34.56)	0.325	−
Pelvic inflammatory disease	34.29 (29.03–39.55)	42.07 (36.46–47.67)	42.63 (37.97–47.30)	0.022	+8.34
Bacterial vaginosis	43.15 (41.83–44.48)	53.73 (48.92–58.54)	44.25 (39.79–48.71)	0.708	−
Acute prostatitis	33.65 (23.03–44.26)	46.84 (36.84–56.83)	31.78 (22.46–41.11)	0.635	−
Percentage of antibiotic beyond duration^d^					
Acute sinusitis (10–14 days)^d^	1.11 (0.03–6.04)	8.00 (3.52–15.16)	0.70 (0.02–3.86)	0.803	−
Acute sore throat (10 days)	3.05 (2.90–3.22)	4.06 (3.78–4.36)	1.94 (1.73–2.16)	0.658	−
Acute cough and bronchitis (5 days)	22.88 (22.15–23.62)	19.79 (18.81–20.80)	19.39 (18.32–20.49)	0.315	−
Community acquired pneumonia (7 days)	11.11 (7.25–16.08)	12.93 (7.42–20.43)	11.98 (7.47–17.89)	0.702	−
Acute otitis media (7–10 days)^d^	5.12 (3.75–6.82)	3.90 (2.65–5.52)	2.61 (1.64–3.92)	0.265	−
Gastroenteritis (5–7 days)^d^	15.65 (15.01–16.31)	8.61 (7.90–9.36)	7.49 (6.74–8.30)	0.026	−8.16
Pericoronitis (3–7 days)^d^	2.48 (1.77–3.36)	2.27 (1.86–2.73)	1.84 (1.51–2.21)	0.333	
Acute periapical abscess (3–7 days)^d^	3.04 (1.32–5.91)	12.07 (4.99–23.30)	−	−	−
Pelvic inflammatory disease (14 days)	1.48 (0.60–3.03)	3.79 (2.64–5.25)	5.36 (4.09–6.89)	0.373	−
Bacterial vaginosis (14 days)	4.87 (4.14–5.68)	2.93 (2.34–3.62)	3.28 (2.73–3.90)	0.516	−
Acute prostatitis (28 days)^d^	1.41 (0.04–7.60)	−	−	−	−
Acute sinusitis (7 days)	15.38 (1.92–45.45)	−	0.71 (0.09–2.56)	−	−

Ratio between B and N = ratio between broad-spectrum and narrow-spectrum antibiotics.

The data were represented as values and 95% CI.

^a^ Test for an overall linear trend for 6 years.

^b^ Change value was the absolute value change of quality indicators during 2010–2011 vs. 2014–2015.

^c^ Referring to the Chinese guidelines, Cellulitis had only 21 first-line antibiotics used during the study period, so it could not calculate its trend change.

^d^ The percentage of oral antibiotics with an exceeded duration of acute sinusitis, acute otitis media, gastroenteritis, pericoronitis, and acute periapical abscess were calculated by using high threshold value, referring to Supplementary Table S1.

### Evaluation of the Quality of Child-Specific Antibiotic Prescribing

**FIGURE 2:**
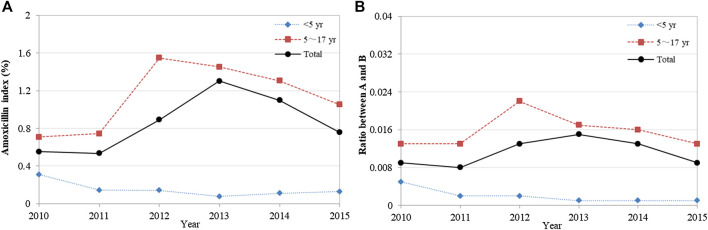
Changes in the quality of child-specific antibiotic prescribing, 2010–2015. **(A)** Amoxicillin index (%); **(B)** Ratio between A and B. Amoxicillin index (%) = Percentage of antibiotics with amoxicillin; Ratio between A and B = Ratio between amoxicillin and broad-spectrum antibiotic.

Analysis of the quality of child-specific antibiotic prescribing showed that the A index and the A/B ratio first increased and then decreased overall, with no statistical difference in trend change (*p* = 0.262 and *p* = 0.539) (Figures 2A,B). These two indicators have been at low levels for a long time, 0.76% and 0.01 by 2015, respectively. Furthermore, amoxicillin use was lower in children aged <5 years than in those aged 5–17 years. Detailed data were shown in Supplementary Table S2.

## Discussion

This study comprehensively evaluated the quality of antibiotic prescribing in primary care facilities in Shenzhen, China. We found that, despite a significant decline in the number of antibiotic used, the B/N ratio increased over time, and the percentages of first-line antibiotics used for most infections and the use of amoxicillin in children were low. These inappropriate antibiotic prescribing might be an important aspect of future antibiotic stewardship in primary care facilities.

In this study, both the overall antibiotic usage and the use of antibiotics for common infections showed a significant downward trend. This was consistent with those reported in previous studies in China (Lin et al., 2016; Li et al., 2019; Li et al., 2020), suggesting that the implementation of antibiotic policies had dramatically reduced the use of antibiotics in primary care facilities. Although the use of antibiotic had declined to a low level, the quality of outpatient antibiotic prescribing was still worth improving in several aspects. First, the use of broad-spectrum antibiotics in most infections of the respiratory, digestive, and genitourinary systems was several times that of narrow-spectrum antibiotics, and this gap had been increasing over time, with bacterial vaginosis in women reaching 18.34 times. This was consistent with the results reported in previous studies that Chinese prescribers tended to favor broad-spectrum antibiotics (Wang et al., 2014; Xiao et al., 2016). Second, the results based on the Chinese guidelines showed that the percentages of first-line antibiotics for 12 common infections were not high, with no more than 50%. And the percentage of oral antibiotics with an exceeded duration for acute cough and bronchitis was still 19.39%. Promoting the appropriate use of antibiotics can be achieved by choosing first-line antibiotics recommended by guidelines when a disease is clearly diagnosed, or avoiding unnecessarily long durations of treatment (Lode, 2014; Spellberg, 2016; Smieszek et al., 2018). Obviously, there is a substantial scope for improving the quality of antibiotic prescribing if prescribers in our study are better adherent to recommended durations of antibiotic treatment. At least, 51,051 days of unnecessary antibiotic could be reduced. Third, for Children’s prescriptions in primary care, although the number of antibiotic used in children aged <5 years declined the most, the B/N ratio was the greatest. Moreover, the use of amoxicillin in children was extremely low, with the A index at 0.76% by 2015. However, this indicator ranged from 30 to 60% in the Netherlands, United Kingdom and Italy (de Bie et al., 2016). Acute respiratory tract infections remained by far the most common indications for children antibiotic prescribing in community practice. Many guidelines recommend amoxicillin as the first-line choice for suspected bacterial respiratory infections, including pneumonia and otitis media (Bradley et al., 2011; Harris et al., 2011; World Health Organization, 2012). For example, amoxicillin became the most highly prescribed antibiotic in pediatric emergency departments and by private practice pediatricians after publication of the new French national guidelines (Ouldali et al., 2017). Appropriate selection of narrow-spectrum antibiotics and encouraging the use of amoxicillin should be an important content of improving the quality of children antibiotic prescribing in China.

Our study suggested that the quality of antibiotic prescribing in Shenzhen’s primary care was not high. This might be due to the following reasons. First, the contents of China’s antibiotic policies were not perfect. The primary objective of these interventions, whether the national antimicrobial stewardship campaign in 2011 or the *Administrative Measures for Clinical Use of Antimicrobial Agents* in 2012, was to control the quantity of antibiotic use. For example, the stewardship campaign required that the percentage of outpatient prescriptions with antibiotics not exceed 20% by the end of 2013. However, there was no strict control requirement for the appropriate type of antibiotic use, such as broad-spectrum antibiotics. Second, although China formulated the *Guidelines for Clinical Application of Antimicrobial Agents in China*, insufficient attention had been paid to the implementation of this guidelines in prescribing behaviors. Because there was basically no research on evaluating the type conformity and time appropriateness of antibiotic prescribing based on Chinese guidelines. Therefore, improving the contents of the antibiotic policy and paying more attention to the practice guidelines, so as to guide the prescriber to prescribe antibiotics correctly, are also the work that cannot be ignored in the antibiotic stewardship.

This study was the first to evaluate in detail the quality of antibiotic prescribing for common infections in primary care facilities in China. Our study selected rich quality indicators, involved many common infections, and carried out the specific evaluations of children. This comprehensive report provided clues to the future antibiotic stewardship from various aspects in China. Second, we used the published Chinese guidelines to evaluate the quality of antibiotic prescribing. This kind of guideline-based evaluation study was of great significance to identify the compliance of prescribers with the guidelines and the rationality of prescribing behaviors. Third, the data of our study were based on the entire prescriptions rather than sampling data, which avoided sampling bias and provided more realistic and reliable assessment. Furthermore, this electronic information system was regularly checked by data inspectors, which ensured the accuracy of the data in this study.

However, our study had several limitations when interpreting the findings. First, this study only carried out the evaluation of prescription quality in primary care facilities in Shenzhen. Further studies with wider coverage and larger sample sizes are needed to evaluate the quality of antibiotic prescribing in China’s primary care. Nevertheless, this study serves as a practical case to provide policy makers and researchers with valuable information on prescription quality. Second, only one primary diagnosis, which was determined by the physician based on a patient’s chief complaint, was documented on our prescription records. It was not clear whether the prescriber decided to replace antibiotics or longer the duration of treatment because of the patient’s other diseases or conditions. Thus, the magnitude of irrational antibiotic use in this regard might be overestimated. Third, we did not get records for culture of infecting pathogens in patients. Therefore, we could not identify whether the antibiotic usage was based on empirical or definitive therapy. We also could not combine patients’ prescription records with laboratory results to determine the objective medication regimen developed by the prescribers. This is difficult because we have limited access to outpatient information. This implied the need to strengthen the standardization and integrity of prescription records in primary care facilities in order to provide more reliable data for prescription evaluation. Fourth, there is a time lag in this study. However, considering that inappropriate use of antibiotics in China’s medical institutions is a long-standing problem, the poor quality of these antibiotic prescriptions is unlikely to be improved significantly. Thus, our results should be still applicable. Furthermore, the comprehensive evaluation model of the quality of antibiotic prescribing in this study can optimize antibiotic stewardship and is worthy of reference in other regions.

## Conclusion

In summary, a practice study in Shenzhen, China, found a significant decrease in the use of antibiotics in primary care facilities. However, the prevalence of broad-spectrum antibiotics in most common infections, the low use of first-line antibiotics, and the poor quality of antibiotic prescribing in children were serious problems. China’s future antibiotic stewardship campaign should be devoted to improving the rationality of antibiotic types and treatment time, and standardizing the content of antibiotic use in children, so as to comprehensively improve the quality of antibiotic prescribing.

## Data Availability

The raw data supporting the conclusions of this article will be made available from the corresponding author on reasonable request.

## Abbreviation

A index, percentage of antibiotics with amoxicillin; A/B ratio, ratio between amoxicillin and broad-spectrum antibiotics; AMR, antimicrobial resistance; B/N ratio, ratio between broad-spectrum and narrow-spectrum antibiotics; CHCs, community health centers; CI, confidence interval, UK, United Kingdom; WHO, World Health Organization.
