# Supplementary material for: Evaluation of the Quality of Antibiotic Prescribing in Primary Care: A Multicenter Longitudinal Study From Shenzhen, China
Source: Front Pharmacol. 2021 Feb 19;11:617260. doi: 10.3389/fphar.2020.617260 (PMC7933578; doi:10.3389/fphar.2020.617260)
Supplement: Supplementary file 1 [file datasheet1.docx]

**Table S1** **First-line antibiotics and duration of antibiotic treatment for** **common infections recommended in the Chinese guidelines**

| **Indications** | **Chinese guidelines** |
| --- | --- |
| Acute sinusitis | 10 to 14 days |
|  | ***First choice:***  Amoxicillin  ***Very unwell or worsening:***  Co-amoxiclav; Co-sulfamethoxazole; First- or second- generation cephalosporins. |
| Acute sore throat | 10 days |
|  | ***First choice:***  Penicillin V and G  ***Penicillin allergy or intolerance:***  Erythromycin; Clarithromycin; First- or second- generation cephalosporins. |
| Acute cough and bronchitis* | 5 days |
|  | ***First choice:***  Amoxicillin; Doxycycline.  ***If amoxicillin and doxycycline contraindicated:***  Clarithromycin. |
| Community-acquired pneumonia* | 7 days |
|  | ***First choice in adults without underlying diseases:***  Amoxicillin; Clarithromycin; Doxycycline. |
| Acute otitis media | 7 to 10 days |
|  | ***First choice:***  Amoxicillin  ***Very unwell or worsening:***  Co-amoxiclav; Co-sulfamethoxazole; First or second generation cephalosporins. |
| Gastroenteritis* | 5 to 7 days |
|  | ***First choice:***  Erythromycin；  ***If erythromycin not tolerated:***  Clarithromycin; Azithromycin; Ciprofloxacin. |
| Pericoronitis | 3 to 7 days |
|  | ***First choice:***  Amoxicillin; metronidazole. |
| Acute periapical abscess | 3 to 7 days |
|  | ***First choice:***  Amoxicillin; metronidazole. |
| Pelvic inflammatory disease | 14 days |
|  | ***First choice:***  Cefotaxime + Doxycycline；  Gentamicin + Clindamycin. |
| Bacterial vaginosis | 14 days |
|  | ***First choice:***  Metronidazole; Tinidazole; Clindamycin; Miconazole. |
| Acute prostatitis | 28 days |
|  | ***First choice:***  Ciprofloxacin; Ofloxacin.  ***after discussion with specialist:***  Levofloxacin; Co-sulfamethoxazole. |
| Cellulitis* | 7 days |
|  | ***First choice:***  Flucloxacillin and continue for further 7 days if slow response.  ***Penicillin allergy:***  Clarithromycin; Clindamycin |

The Chinese guidelines = the *Guidelines for Clinical Application of Antimicrobial Agents in China* *(2004 edition).*

* The antibiotic treatment recommended by Public Health England (PHE) guidance during 2013-2015.

**Table S2 Change in the quality of overall antibiotic prescribing, 2010-2015**

| **Characteristics** | **2010** | **2011** | **2012** | **2013** | **2014** | **2015** | ***P* for overall linear trend ^a^** |
| --- | --- | --- | --- | --- | --- | --- | --- |
| **Antibiotics/ per 100 consultations** | 93.50 | 89.44 | 50.94 | 28.06 | 24.80 | 19.98 | 0.004 |
| Age group, years |  |  |  |  |  |  |  |
| <5 | 85.47 | 85.36 | 41.27 | 11.93 | 10.70 | 10.37 | 0.009 |
| 5-17 | 93.66 | 91.24 | 43.28 | 24.44 | 24.11 | 19.71 | 0.010 |
| ≥18 | 87.01 | 89.48 | 52.26 | 29.55 | 25.99 | 21.29 | 0.005 |
| **Ratio between B and N** | 1.39 | 1.53 | 1.97 | 3.34 | 2.88 | 4.84 | 0.009 |
| Age group, years |  |  |  |  |  |  |  |
| <5 | 2.21 | 3.39 | 2.02 | 70.61 | 48.21 | 106.27 | 0.020 |
| 5-17 | 1.82 | 1.88 | 3.39 | 7.99 | 7.77 | 13.41 | 0.005 |
| ≥18 | 1.31 | 1.42 | 1.87 | 2.73 | 2.27 | 3.61 | 0.010 |
| **Percentage of first-line antibiotics** | 26.42 | 27.97 | 29.04 | 38.51 | 34.54 | 34.91 | 0.054 |
| Age group, years |  |  |  |  |  |  |  |
| <5 | 29.15 | 32.46 | 44.73 | 63.04 | 42.25 | 46.21 | 0.215 |
| 5-17 | 26.23 | 32.91 | 37.67 | 44.55 | 36.63 | 42.73 | 0.053 |
| ≥18 | 26.33 | 27.47 | 27.8 | 36.73 | 33.96 | 33.15 | 0.065 |
| **Percentage of oral antibiotics beyond duration** | 11.99 | 8.91 | 12.15 | 13.27 | 12.67 | 13.38 | 0.181 |
| Age group, years |  |  |  |  |  |  |  |
| <5 | 13.16 | 7.54 | 4.74 | 8.26 | 12.99 | 8.23 | 0.884 |
| 5-17 | 14.57 | 7.1 | 11.57 | 6.73 | 11.35 | 5.99 | 0.265 |
| ≥18 | 11.76 | 9.12 | 12.79 | 15.08 | 12.91 | 15.28 | 0.097 |
| **Amoxicillin index (%)** | 0.553 | 0.534 | 0.892 | 1.303 | 1.098 | 0.756 | 0.262 |
| Patient age (years) |  |  |  |  |  |  |  |
| <5 | 0.311 | 0.144 | 0.142 | 0.077 | 0.111 | 0.130 | 0.121 |
| 5-17 | 0.707 | 0.744 | 1.547 | 1.453 | 1.305 | 1.051 | 0.320 |
| **Ratio between A and B** | 0.009 | 0.008 | 0.013 | 0.015 | 0.013 | 0.009 | 0.539 |
| Patient age (years) |  |  |  |  |  |  |  |
| <5 | 0.005 | 0.002 | 0.002 | 0.001 | 0.001 | 0.001 | 0.042 |
| 5-17 | 0.013 | 0.013 | 0.022 | 0.017 | 0.016 | 0.013 | 0.910 |

The ratio between B and N= the ratio between broad-spectrum and narrow-spectrum antibiotics;

The ratio between A and B= the ratio between amoxicillin and broad-spectrum antibiotics

a *P* values were associated with linear regression analysis.
